# Investigating the activation of passive metals by a combined in-situ AFM and Raman spectroscopy system: a focus on titanium

**DOI:** 10.1038/s41598-023-33273-1

**Published:** 2023-04-14

**Authors:** L. Casanova, M. Menegazzo, F. Goto, M. Pedeferri, L. Duò, M. Ormellese, G. Bussetti

**Affiliations:** 1 Department of Chemistry, Materials and Chemical Engineering “G. Natta”, Politecnico di Milano, Via Mancinelli 7, 20131 Milano, Italy; 2 Department of Physics, Politecnico di Milano, Piazza Leonardo Da Vinci, 20133 Milano, Italy

**Keywords:** Engineering, Electrochemistry, Chemistry, Surface spectroscopy, Physics, Raman spectroscopy, Materials science, Metals and alloys

## Abstract

Understanding the main steps involved in the activation of passive metals is an extremely important subject in the mechanical and energy industry and generally in surface science. The titanium-H_2_SO_4_ system is particularly useful for this purpose, as the metal can either passivate or corrode depending on potential. Although several studies tried to hypothesise the surface state of the electrode, there is no general consensus about the surface state of Ti in the active–passive transition region. Here by combining in-situ atomic force microscopy (AFM) and Raman spectroscopy, operating in an electrochemical cell, we show that the cathodic electrification of Ti electrodes causes the dissolution of the upper TiO_2_ portion of the passive film leaving the electrode covered by only a thin layer of titanium monoxide. Fast anodic reactions involved the acidification of the solution and accumulation of sulphur containing anions. This produces a local increase of the solution turbidity, allowing to distinguish favourable regions for the precipitation of TiOSO_4_·2H_2_O. These results give a clear answer to the long-stated question of the physical origin behind the formation of negative polarization resistances, sometimes occurring in corroding systems, and a rationale about the proton-induced degradation of passive surfaces in presence of sulphur containing species.

## Introduction

A physical–chemical system constituted by an activating electrode, like titanium Gr. 2 immersed in sulphuric acid, offers the opportunity of studying an electrochemical interface characterised by spatial inhomogeneities and time dependent reactions responsible of multiple steady states. The importance of studying the dissolution process of Ti in sulphuric acid was highlighted in the previous works^1–6^, regarding the complex electrochemical interface developing over the metal, as a result of the multitude possible valence states assumed by the metal cation and the formation of complexes from the coupling of Ti cations and S bearing anions. Titanium continues to deserve attention^7^ as it is a strategic metal particularly employed, for example, in the aerospace industry where it comes in contact with sulphuric acid when airplanes flight in the troposphere and stratosphere^8^. This causes de-passivation to occur upon the reduction of its oxide layer, a process still far to be completely understood^9,10^. Here the attack can be very dangerous when the metal is employed, for example, in the construction of fan blades for military engines. This can severely affect the creep resistance and the components capability to sustain the high load condition. Therefore, understanding the main steps leading to titanium activation can be of interest to put the basis for future mitigation strategies involving alloying or surface coatings^11,12^. This could be done exploiting specific in-situ analysis combining topographic and surface spectroscopic acquisitions while controlling the electrochemical reactions by a potentiostat. For example, reduced titanium oxide phases, developed upon cathodization, are quite sensitive to atmospheric oxygen so that their investigation should involve the protecting effect of a strong reducing environment like concentrated sulphuric acid, to preserve their stoichiometry. Following this idea, the reader is advised to compare the Raman spectrum, collected in deionised water, (Supplementary Fig. 1) of the air exposed concentration cell with the in-situ analysis of Fig. 3f–k.

A Ti electrode, immersed inside a reducing acidic solution, allows the corrosion potential (E_corr_) to fall in correspondence of values around − 300 mV/SCE (Saturated Calomel Electrode), where the oxide is considered to be almost proton transparent^4,13^. From a phenomenological point of view, three tentative models have been developed to account for the surface state of the material during the active to passive transition, hypothesising (i) the formation of a *monolayer of adsorbed species*, (ii) the presence of a *reduced oxide* or (iii) an interface characterised by a *mix* of both species^4^. Here, we definitively prove that, in the transition region, the Ti surface is always characterised by a thin oxide mainly composed by TiO and located between passivity and the critical electrochemical potential (E_crit._), confirming the validity of the second hypothesis previously advanced. In these regions, fast anodic kinetics followed by cations hydrolysis implies the local acidification of the electrode surface, resulting at least in the decrease of one unit in solution pH. According to electroneutrality, those regions are affected by high accumulation of sulphates and bisulphates forming a supersaturated concentration cell, having diameters of tens of micrometres that evolves with the process. Here, the involved chemical-electrochemical reactions lead to the precipitation of a film of TiOSO_4_·2H_2_O.

## Methods

**Figure 1:**
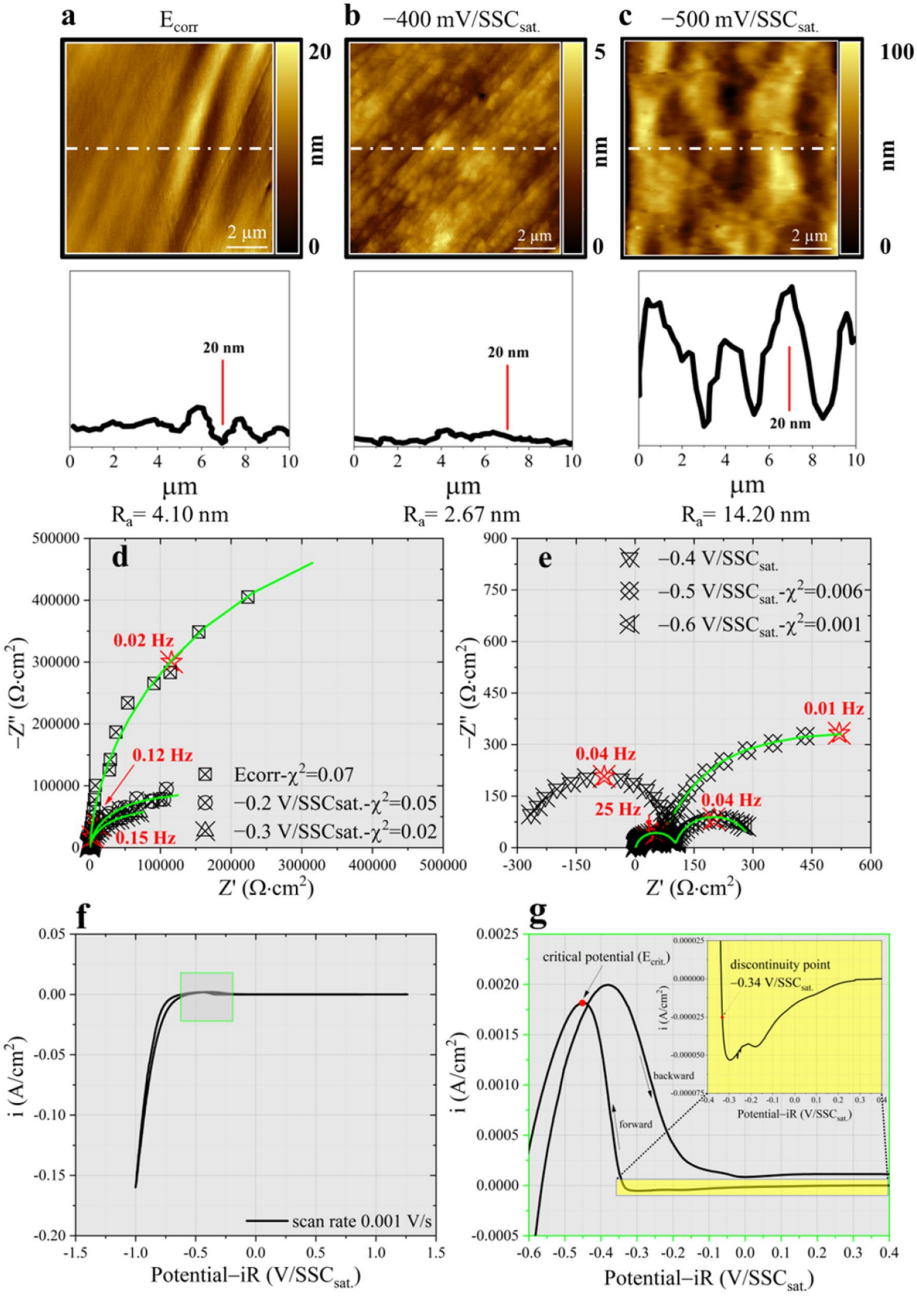
Topographic and electrochemical investigation. (**a**) image acquired at E_corr_. (**b**) image acquired at − 0.4 V/SSC_sat._. (**c**) image acquired at − 0.5 V/SSC_sat._. (**d**) Nyquist representation of impedance data evaluated at: E_corr_, − 0.2 V/SSC_sat._ and − 0.3 V/SSC_sat._. (**e**) Nyquist representation of impedance data evaluated at: − 0.4 V/SSC_sat._, − 0.5 V/SSC_sat._ and − 0.6 V/SSC_sat._ (**f**) and (**g**) cyclic voltametric experiment performed on Ti Gr. 2 immersed in 40 %v/v H_2_SO_4_.

All the experiments are performed over 1 cm^2^ circular titanium Gr. 2 (UNS R50400) samples cut by metal shearing whose chemical composition is as follows: Fe (0.30%) + O (0.25%) + C (0.08%) + N (0.03%) + H (0.015%) + Ti (balance) supplied by RL3 S.r.l.. Samples are mechanically polished with silicon carbide papers and alumina particles to obtain a mirror like surface. Before the electrochemical experiments, samples are cleaned in ultrasound with acetone and then washed in deionised water. Titanium is always allowed to passivate for 24 h air exposure in order to saturate the oxide thickness^9^. According to the literature, a layer ~ 5 nm thick composed by a mix of 2 +, 3 + and 4 + oxidation states is expected^9^. All the electrochemical tests are performed using a Metrohm Autolab PGSTAT equipped with a FRA32M module for EIS using a 4 ml PTFE 3 electrode cell (ASTM G5^14^) with a platinum wire used as a pseudo-reference and counter electrode. A pseudo-reference is preferred to avoid any solution contamination. Values are then converted with respect to a saturated silver-silver chloride (+ 0.197 V/SHE) reference electrode (SSC_sat._). The experiment is repeated for a total of 10 times in an electrolytic solution of 40 %v/v H_2_SO_4_ (7.46 M, Merck 99.999%) at room temperature (21 °C). The impedances are evaluated at fixed potential values: at the corrosion potential in passive condition E_corr_ (~ + 0.15 V/SSC_sat._), at − 0.2 V/ SSC_sat._, at − 0.3 V/ SSC_sat._, below the conduction band (E_cb_) edge − 0.4 V/ SSC_sat._, near the critical potential − 0.5 V/SSC_sat._ and in the active region − 0.6 V/SSC_sat._. All the EIS acquisitions are performed after 30 min of stabilisation both in free corrosion and after the application of a potential, in a frequency window between 10^−2^ to 10^5^ Hz, collecting 10 points per decade with a voltage amplitude, of the sinusoidal perturbation, of 10 mV_rms_ considering no repetition for each data point acquired by the system. The procedure can be better visualised in Fig. 1 where a cyclic voltametric experiment, carried out with a scan rate of 0.001 V/s, presents the potential window and the kinetics of interest. In-situ electrochemical atomic force microscopy (EC-AFM) and in-situ Raman spectroscopy acquisitions are performed in correspondence of previously mentioned potential values. EC-AFM is performed using a commercial NTEGRA Spectra set-up (NT-MDT). Images are collected in-situ (acquisition time ~ 1000 s), in non-contact mode (υ_0_ ~ 130 kHz) by employing VIT P/IR tips (TipsNano). Raman spectroscopy is performed by employing an excitation laser source at 532 nm, having a power of 5 mW. Quantification of sulphate molarities of the uncontaminated solution is afforded considering pure solutions of sodium sulphate (see Supplementary Fig. 2 and Table 1 for details). Crystal structure is characterized by x-ray diffraction using a Philips PW3020 goniometer with Cu K_α1_ radiation (1.54058 Å) in Bragg–Brentano geometry. X-ray photoelectron spectroscopic (XPS) experiments is performed using a non-monochromatized Mg-Kα x-ray source (hν = 1253.6 eV) and the photoelectron kinetic energy is measured by a 150 mm hemispherical analyser (PHOIBOS150) from SPECS^15^. Core level regions are acquired with pass energy of 20 eV, with energy resolution of 1 eV. The base pressure of the vacuum chamber during XPS experiments was $3\cdot {10}^{-10} \mathrm{Torr}$ and the sample is transferred from the EC cell to the vacuum system in Ar atmosphere to prevent surface contamination and reoxidation by air exposure. Considering that a reduction of reflectivity (surface roughness) decreases the signal-to-noise ratio and precludes the acquisition of significant data, all the in-situ analysis are carried out only along the descendent potential sweep before the activation of the electrode.

## Results and discussion

### Proton-induced degradation of the upper TiO_2_ layer

**Figure 2:**
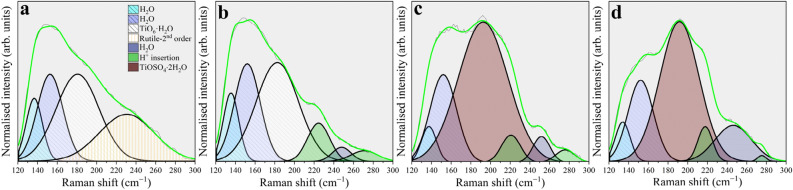
Low frequency Raman spectroscopy analysis. (**a**) Raman spectrum acquired at E_corr_. (**b**) Raman spectrum acquired at − 0.4 V/SSC_sat._. (**c**) Raman spectrum acquired at − 0.5 V/SSC_sat._. (**d**) Raman spectrum acquired at − 0.6 V/SSC_sat._.

Once a cathodic potential is applied to the electrode, the Fermi level of the oxide, covering the metal, is raised favouring the reduction of protons, the flow of a cathodic current and degradation of the upper layers. Figure 1a–c, which explore an area of (10 × 10) µm^2^, reports the topographical evolution of the electrode surface as a consequence of the modification induced by the cathodic sweep applied to the polycrystalline Ti substrate. While AFM images at E_corr_ (Fig. 1a) were acquired immediately, ~ 2.5 h are required to induce the surface modifications seen in Fig. 1b and ~ 4 h for the ones of Fig. 1c. The reader is referred to Supplementary Fig. 3 for comparing the nanometric modification seen in Fig. 1a–c and Supplementary Fig. 4, induced by the corrosion process, with the topography of the pristine sample. In Fig. 1a, it is possible to see that, once the sample is in free corrosion and up to potentials above E_cb_, the pristine morphology is preserved: longitudinal ripples are consequence of the surface mechanical preparation. This result is in agreement with another study^16^, which demonstrates that dissolution initially occurs between grains and evolves in the formation of multitude local peaks as highlighted in Fig. 1b. The cathodic wave observed in the insert of Fig. 1g near − 0.18 V/SSC_sat._^17^ is assigned to the redox couple Ti^4+^/Ti^3+^ while the one at − 0.3 V/SSC_sat._^18^ to the flat band potential of TiO_2_. Those waves prove that proton insertion occurred also above E_cb_ (~ − 0.34 V/SSC_sat._^19–22^), resulting in the substantial degradation of the upper TiO_2_ layer. An additional confirmation comes from in-situ Raman spectroscopy analysis: (1) the rutile 2nd order feature at ~ 230 cm^−1^ of Fig. 2a progressively redshifts and decreases in the integrated signal intensity; (2) a new peak at 218 cm^−1^ (Fig. 2b) appears and it is attributed to the H_3_O^+^ insertion inside the octahedral arrangement of the Ti and O ions^23,24^; (3) the E_g_(symmetric stretching)-rutile (444 cm^−1^) and A_1g_(anti-symmetric bending)-rutile (614 cm^−1^) modes disappears (see Supplementary Fig. 5 and Table 2) and (4) a peak rises at 271 cm^−1^ (Fig. 2b). The latter could be related to the E_g_ of Ti_2_O_3_ or to the vibrational mode, as found by Tao et al*.*^25,26^, occurring upon proton insertion in titanate structures.

Apart from oxide related lines, the spectra highlighted in Fig. 2 are characterised by the presence of peaks belonging to vibrational modes related to H bonds present among H_2_O molecules, for further details the reader should refer to Table 3. Knowledge about those features is gained by acquiring Raman spectra during cathodization over a pure Au substrate in the tested solution (see Supplementary Figs. 6, 7 and Table 4). Oxide reduction is confirmed by the sudden decrease of the arc diameter of Fig. 1d (see also Supplementary Fig. 8), indicative of the charge transfer resistance (R_ct_) that passes from 1.09·10^6^ Ω·cm^2^ at E_corr_ to 8.46·10^4^ Ω·cm^2^ at − 0.2 V/SSC_sat._.

Consequently, the capacitance of the system^27^ increases from 13.9 µF/cm^2^ to 16.8 µF/cm^2^, denoting charge accumulation in the space charge region at the semiconductor-electrolyte interface. The above process description can be summarised in Eq. (1), which describes the process of proton insertion coupled by electron transfer.

1 $${Ti}^{4+}{O}_{2}+{e}^{-}+{H}_{3}{O}^{+}\leftrightarrow Ti\left(O\right)\left(OH\right)+{H}_{2}O$$

With the increase of the charge transfer reaction kinetics, the system becomes partially controlled by mass transport^28^. For fitting purpose, of the impedance spectra, semi-infinite diffusion is considered (Supplementary Fig. 9 and Table 5 collecting fitting result). Given the high acidity it is reasonable to assume mass transport to be mainly related to solid state diffusion of protons inside the TiO_2_ lattice^21^ obtaining diffusivities^29,30^ (Eq. (1) in Supplementary material) equal to ${D}_{{H}_{3}{O}^{+}}^{-0.2 V/SSCsat.}=3.10\cdot {10}^{-18} \frac{{cm}^{2}}{s}$ and ${D}_{{H}_{3}{O}^{+}}^{-0.3 V/SSCsat.}=6.67\cdot {10}^{-18} \frac{{cm}^{2}}{s}$, entailing a proton penetration (Eq. (2) in Supplementary material) of 0.97 nm at − 0.2 V/SSC_sat._ and 2.01 nm at − 0.3 V/SSC_sat._ according to present polarization periods. This is purely speculative but we denote a certain similarity between diffusion length extracted by EIS and the R_a_ parameter obtained by EC-AFM, an hypothesis deserving further investigation in future researches. Having obtained titanium oxide hydrogenation, via an electrochemical route, this induces the formation of shallow donor defects, lying immediately below E_cb_^31^. Under those circumstances the specie moves interstitially forming almost covalent bond with lattice O without interacting with O vacancies^31^.The oxide protectiveness vanishes below E_cb_, where the current changes from cathodic to anodic, inducing substantial topological modifications (Fig. 1b).

This fact highlights that the kinetic of the process is dictated by the oxidation of the metal cation as $Ti\to {Ti}^{3+}+3{e}^{-}$. In the literature, this is generally attributed to the fact that, below E_cb_, the phase oxide ceases to exist leading to the formation of an adsorbed monolayer^4^. Conversely, our study demonstrates this assumption to be partially wrong, because a thin reduced oxide layer always covers Ti in the transition region.

**Figure 3:**
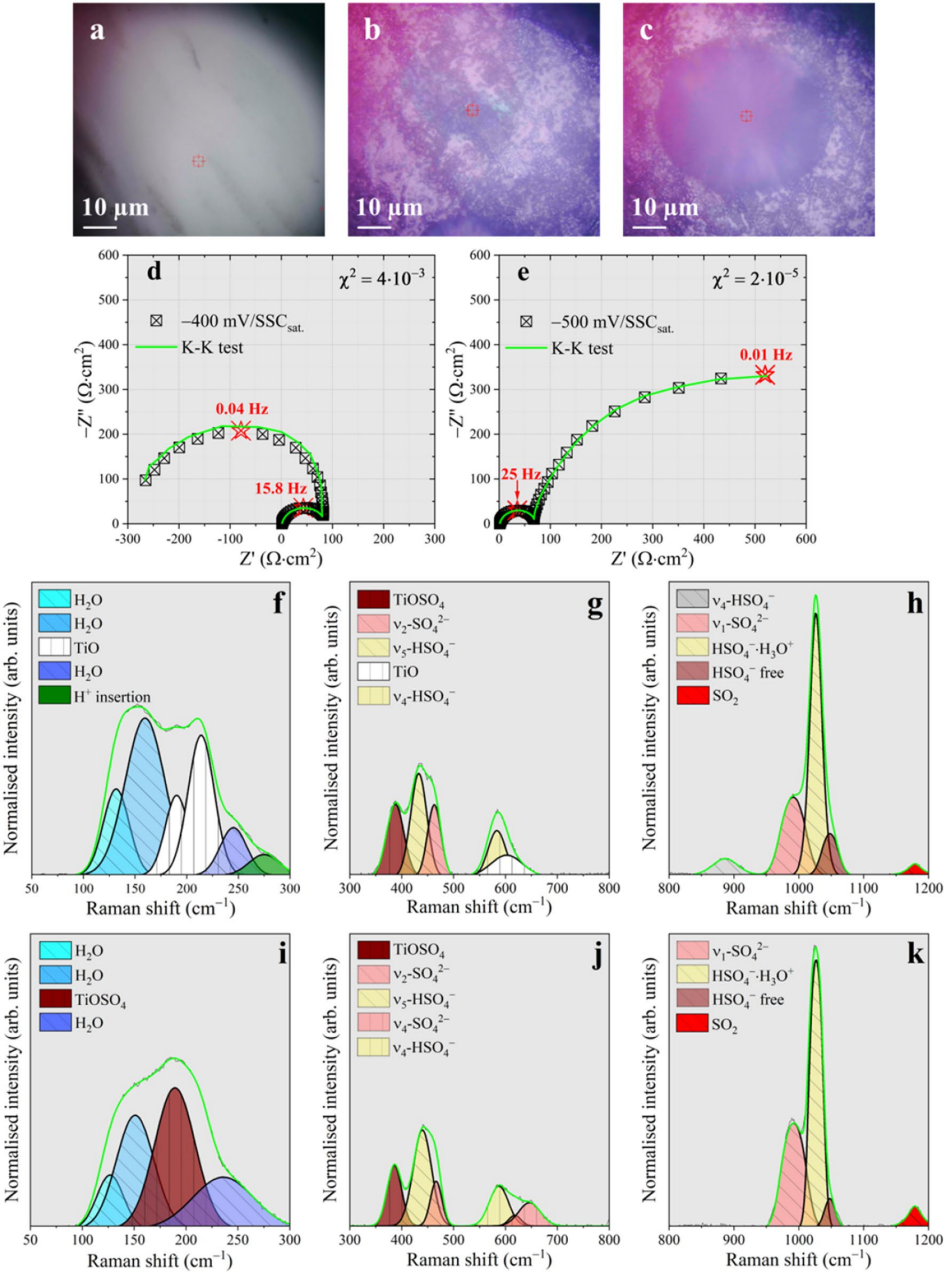
Investigation of the corrosion triggering mechanism. (**a**) Optical image of uncorroded sample. (**b**) optical image showing fluorescence due to oxide reduction. (**c**) Optical image showing formation of a concentration cell. (**d**) Nyquist plot collected in correspondence of image b. (**e**) Nyquist plot collected in correspondence of concentration cell formation. (**f**) low frequency Raman spectrum extracted in correspondence of the fluorescent region seen in image b. (**g**) 300–800 cm^−1^ Raman spectral portion collected inside the fluorescent region. (**h**) 800–1200 cm^−1^ Raman spectral portion collected inside the fluorescent region. (**i**) low frequency Raman spectrum extracted in correspondence of the concentration cell seen in image c. (**j**) 300–800 cm^−1^ Raman spectral portion collected inside the concentration cell seen in image **c**. (**k**) 800–1200 cm^−1^ Raman spectral portion collected inside the concentration cell seen in image c.

In-situ surface analysis with an optical microscope (Fig. 3a–c) reveals the presence of fluorescent spots randomly distributed on the sample surface when the latter is polarised below − 0.4 V/SSC_sat._. Colour centres over titanium oxide are generally the result of the local oxide reduction with formation of electronic defects. This local dissolution may arise from regions of high electronic conductivity^32^. Keeping this in mind and focusing on EIS (Fig. 3d and e), the Nyquist representation shows the occurrence of a low frequency capacitive loop with negative real impedances (negative curl). This apparently strange behaviour, also observed during anodic dissolution of iron in sulphuric acid^33,34^, does not violate the principle of linearity, stability and causality giving consistency to the EIS results^35^.

Here, we point out that the negative polarization resistance, in the Nyquist representation and in polarization plots is consequence of the enlargement of the fluorescent regions (FR), affected by the dissolution of the protective upper TiO_2_ layer (see below), causing a progressively increasing anodic current in opposition to the decreasing anodic potential. The phenomenon develops in a time scale compatible with the low frequency spectrum mainly affecting the zero frequency value of the impedance, i.e., the polarisation resistance. Raman analysis localised on FR (Fig. 3f–k) shows the presence of clear components at 187 (Fig. 3f), 215 (Fig. 3f) and 603 cm^−1^ (Fig. 3g), whose positions and reciprocal intensities perfectly agree with the TiO reference spectrum^36^. The TiO_2_ upper layer is thus locally degraded, leaving the lower unprotective TiO structure exposed to the solution.

### Formation of the concentration cell

**Figure 4:**
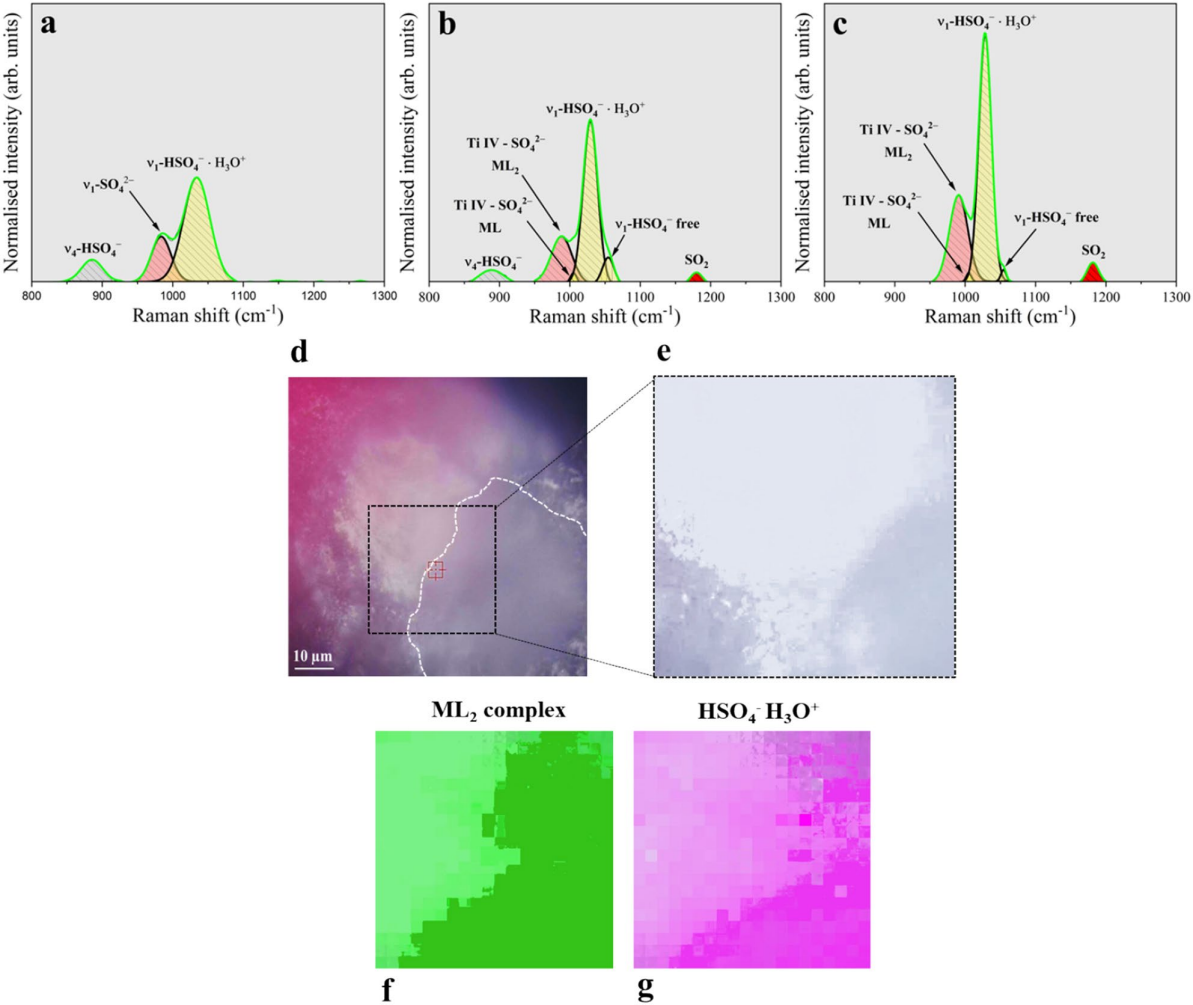
Analysis of the concentration cell by Raman spectroscopy. (**a**) Raman spectrum of the 800–1200 cm^−1^ region collected outside the concentration cell. (**b**) Raman spectrum of the 800–1200 cm^−1^ region collected at the border of the concentration cell. (**c**) Raman spectrum of the 800–1200 cm^−1^ region collected at the centre of the concentration cell. (**d**) Optical image collected in correspondence of a concentration cell delimited by the white dotted profile. (**e**) enlarged view of image d. (**f**) Raman map of the intensity of the 991 cm^−1^ component of the ML_2_ complex. (**g**) Raman map of the intensity of the 1029 cm^−1^ component of the HSO_4_^−^·H_3_O^+^.

Previously highlighted steps result, at the end, in macroscopic surface modifications of the electrode. However, it will be shown that the opportunity offered by the present experimental apparatus is to capture the very first instant of formation of local phenomena responsible for the metal activation. Fast anodic currents are expected to result in feeding those unprotected regions that are affected by the dissolution of the upper TiO_2_ layer. Since Ti^3+^ is a strong reducing agent, it is reasonable to assume this ion to be readily reactive with one coordinated water molecule to form Ti^4+^. As a result, Ti^4+^ ions can readily hydrolyse leading to local acidification^37^. This can be demonstrated, electrochemically, according to the cyclic voltammetry reported in Fig. 1f. Here the shift (~ 70 mV) of the anodic peak in the positive direction is consistent with the pH decrement of one unit^4^. This positive surface charge is compensated by an anion accumulation (SO_4_^2−^ and HSO_4_^−^) as seen by the enhancement of the Raman lines intensity in the spectral region above 800 cm^−1^ (see Fig. 4). The main features are assigned to the stretching mode of the bisulphate (ν_1_-HSO_4_^−^ at ~ 1034 cm^−1^)^38–40^ and sulphate (at ~ 980 cm^−1^)^41–43^.

The Raman shift found for the former line is indicative of protons pairing. This chemical state almost completely substitutes the free bisulphate ion inside the concentration cell, as confirmed by the disappearance of the ν_4_-HSO_4_^−^ line found generally around 890 cm^−1^. Here, there is a particular increment in the solution turbidity, denoting the formation of a circular concentration cell as in Fig. 3c. In this regions, Raman features (Fig. 3i,j) resemble the spectra acquired over the electrode surface when the potential is swept below − 0.5 V/SSC_sat._ (Fig. 2c and d). At those cathodic potentials, the surface appears black coloured by the presence of a corrosion product. The latter can be characterised by AFM (Fig. 1c), which detects an increase of the R_a_ (14.2 nm). The increase of surface coverage by the corrosion product contributes to the pinch-off of the anodic current, resulting in positive R_p_.

The deposit are characterised by photoemission experiments and x-ray diffraction (Supplementary Fig. 10) indicating TiOSO_4_·2H_2_O as the main constituent. The Raman lines at 192 cm^−1^ and 391 cm^−1^, collected inside the concentration cell and in regions covered by the black corrosion product, coincide with the vibrations of the TiO_6_ octahedra composing an oxy-sulphate crystal structure^44^. Moreover, depending on the hydration level of the sulphonated film doublet and triplet may arise from the ν_2_-SO_4_^2−^ and ν_4_-SO_4_^2−^ vibrational mode according to the symmetry lowering occurring when passing from the liquid to the solid state^41^.

### Precipitation of the corrosion product

A detailed Raman spectroscopic investigation of the concentration cell and its neighbouring regions can favour the understating of the chemical-electrochemical steps leading to the precipitation of the corrosion product. Some authors^45,46^ found no precipitation of hydrated TiOSO_4_ until the concentration of H_2_SO_4_ reached 65 wt.%, quite far from the nominal concentration employed in the present work, confirming the suitability of the model involving the formation of the concentration cells. There is further evidence of a lower local pH, triggering concentration cell formation, according to the redshifts (1029 cm^−1^, Supplementary Table 6) of the HSO_4_^−^·H_3_O^+^ stretching line, resulting from paring with protons^47,48^. Raman quantification process indicates a bulk solution characterised by 1.23 M of sulphates and 6.23 M of bisulphates according to literature^49^. The speciation of previous anions with Ti^4+^ considerably modified previous numbers, creating favourable conditions for corrosion product deposition. In particular, inside the concentration cell there is evidence of formation of two Ti IV-sulphates complexes, found in literature to be bidentate (ML_2_—991 cm^−1^) and unidentate systems (ML_1_—1010 cm^−1^)^38,39,50^. This is the result of their considerably higher stability constant found for sulphate complexes with respect to bisulphate systems^37,38,51,52^. The larger integrated intensity found for the ML_2_ complex, inside the concentration cell, allows to hypothesise the establishment of a larger sulphate to Ti IV concentration ratio (Fig. 4d). We have found substantial changes in the integrated intensity of peaks related to the main ions checking different regions outside and inside the concentration cell. In particular, at the periphery of the concentration cell, sulphate species start to complex with Ti^4+^ with a progressively increasing trend as the centre of the concentration cell is approached. Analysis of the stretching vibration of bisulphates allows to see that the overall integrated intensity is consistently reduced according to the strong decrease of peak width at the full width at half maximum (FWHM) (from ~ 46 cm^−1^ outside the concentration cell to ~ 15 cm^−1^ inside the latter). In these regions, the strong modifications seen at the previous bisulphate ion line are assumed to be related to the interaction with the electrode surface, according to the electron transfer reaction described by Eq. (2). This is capable to explain the bisulphate concentration decrement inside the concentration cell. This reaction is energetically favoured for potentials more cathodic than 0.16 V/SHE in acidic solutions.

2 $$HS{O}_{4}^{-}+{H}^{+}+2{e}^{-}\leftrightarrow S{O}_{2}+2{H}_{2}O$$

This contributes to the accumulation of SO_2_ (see Raman peak at ~ 1180 cm^−1^ inside the concentration cell). According to Eq. (3), SO_2_ may react with water and give SO_4_^2−^, thus providing the excess of sulphate species necessary to complex with Ti^4+^.

3 $$S{O}_{2}+2{H}_{2}O\leftrightarrow S{O}_{4}^{2-}+4{H}^{+}+2{e}^{-}$$

This mechanism gives a rationale of the corrosion products precipitation and the formation of a deposit according to a polymerisation reaction^46^. In conclusion, it is demonstrated how the activation of a Ti Gr. 2 electrode starts in local weak spots where the dissolution of the upper TiO_2_ layer results in the formation of fluorescent regions characterised by a greenish hue. Here, the fast anodic kinetic is responsible of the local acidification resulting from cations hydrolysis. Consequent charge compensation involves the migration of S containing anions, entailing the formation of growing concentration cells visually distinguished from the background according to an enhanced solution turbidity. Reaction between Ti^4+^ ions and SO_4_^2−^ implies the formation of unidentate and bidentate complexes which, according to a polymerisation reaction, promote the precipitation of a corrosion product. This in-situ investigation confirms those regions to be the preferential zones affected by the precipitation of TiOSO_4_·2H_2_O, demonstrating Ti to be always covered by a titanium monoxide layer surmounted by an oxy-sulphate corrosion product before complete activation of the electrode is reached.

## Supplementary Information

Supplementary Information.

## Data Availability

The datasets generated and/or analysed during the current study are available in the Supplementary material.
