# Supplementary material for: Investigating the activation of passive metals by a combined in-situ AFM and Raman spectroscopy system: a focus on titanium
Source: Sci Rep. 2023 Apr 14;13:6117. doi: 10.1038/s41598-023-33273-1 (PMC10104870; doi:10.1038/s41598-023-33273-1)
Supplement: Supplementary file 1 — Supplementary Information. [file 41598_2023_33273_MOESM1_ESM.docx]

**Investigating the activation of passive metals by a combined in-situ AFM and Raman spectroscopy system: a focus on titanium**

L. Casanova^a^*, M. Menegazzo^b^, F. Goto^b^, M. Pedeferri^a^, L. Duò^b^, M. Ormellese^a^ and G. Bussetti^b^

*^a^Dept. of Chemistry, Materials and Chemical Engineering “G. Natta”, Politecnico di Milano, Via Mancinelli 7, 20131 Milano, Italy*

*^b^Dept. of Physics, Politecnico di Milano, Piazza Leonardo Da Vinci, 20133 Milano, Italy*

**Fig. 1: Raman spectrum of the concentration cell collected in water.**

*
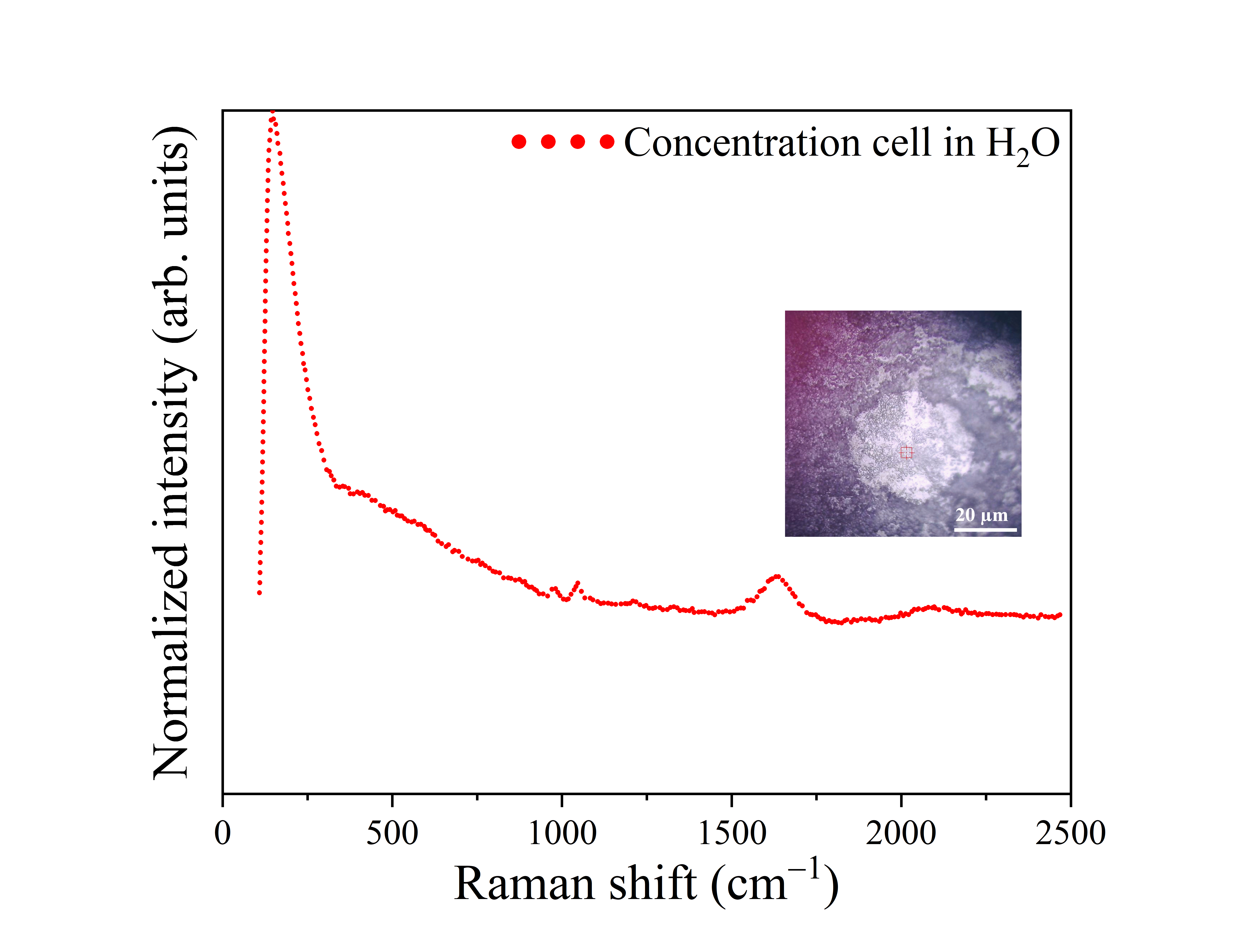
*

Complete survey of the Raman spectrum of the concentration cell collected in deionised water.

**Fig. 2: regression analysis to obtain the molar intensity coefficient of the ν_1_-SO_4_^2−^ line.**

**
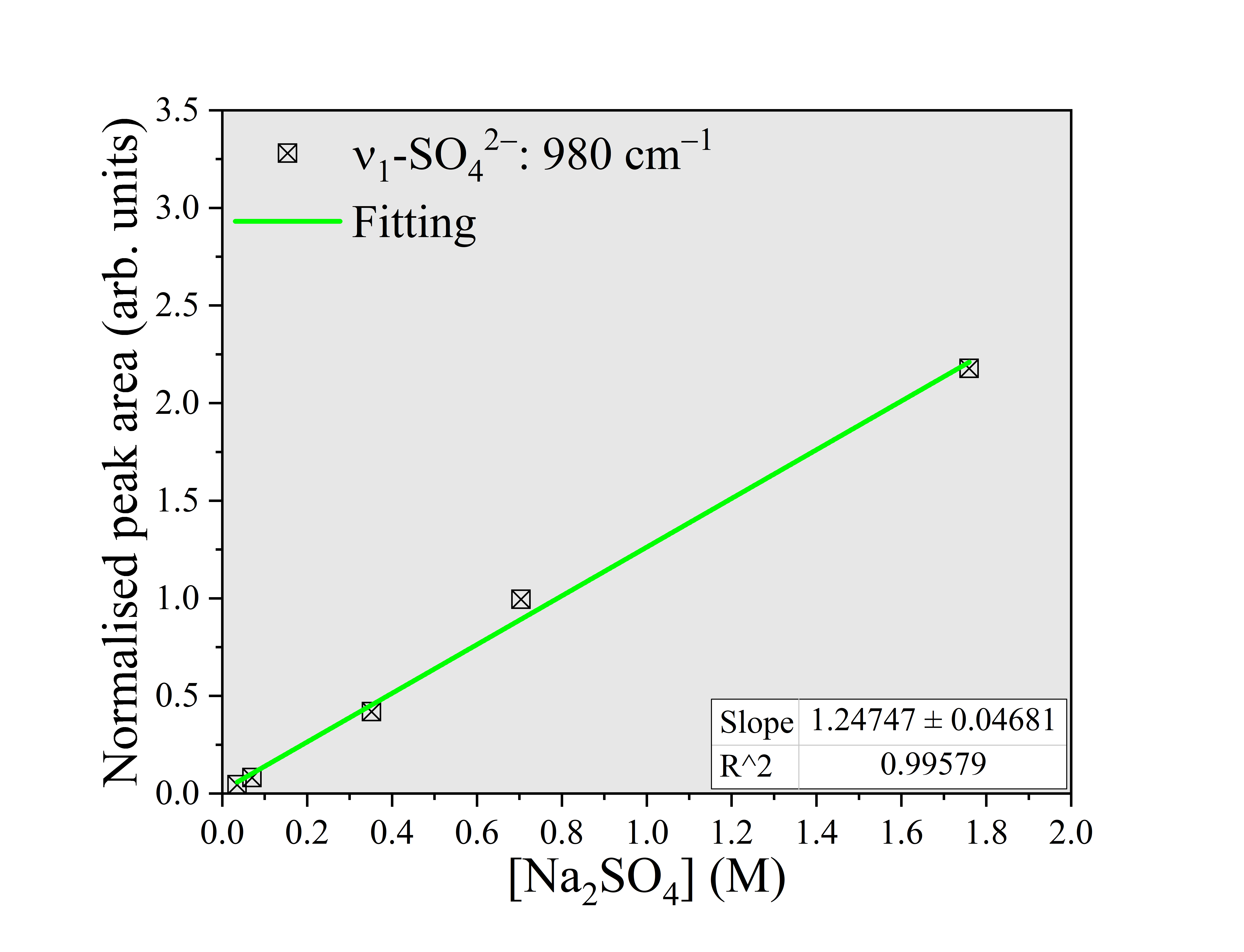
**

Calibration curve of the symmetric stretching line of sulphates (980 cm^−1^) for various sodium sulphate concentrations.

**Fig. 3: AFM image of pristine Ti Gr. 2 collected in air.**


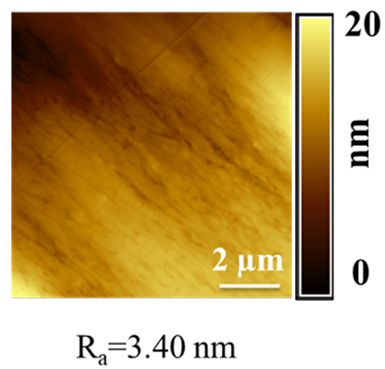


Complete AFM image collected in air before corrosion tests.

**Tab. 1: Raman peaks of 250 g/l Na_2_SO_4_ tested on a pure gold substrate.**

| **Peak position (cm^−1^)** | **FWHM (cm^−1^)** | **Area %** | **Assignment** | **References** |
| --- | --- | --- | --- | --- |
| 448 | 41 | 8.26 | ν_2_-SO_4_^2−^ | ^23^ |
| 615 | 43 | 5.91 | ν_4_-SO_4_^2−^ | ^23^ |
| 978 | 30 | 58.69 | ν_1_-SO_4_^2−^ | ^23^ |
| 1110 | 63 | 9.55 | ν_3_-SO_4_^2−^ | ^23^ |
| 1611 | 90 | 17.29 | bending of H_2_O | ^32^, ^33^, ^34^ |

**Fig. 4: AFM images of titanium acquired in 40 %v/v H_2_SO_4_.**

**
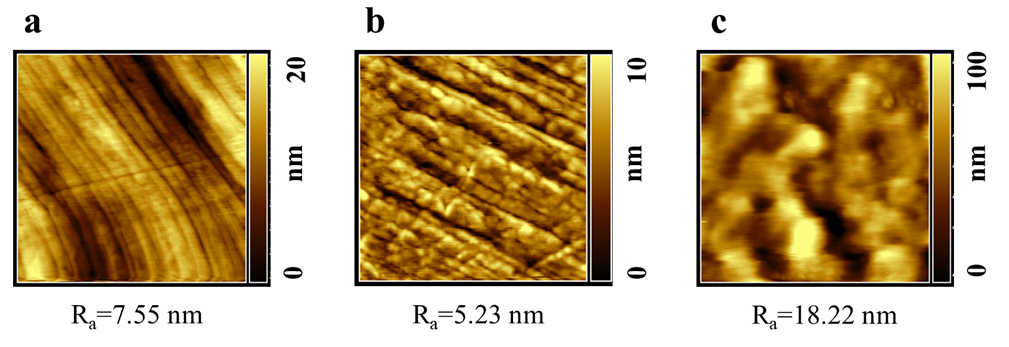
**AFM image collected at **a** E_corr_, **b** −400 mV/SSC_sat._ and **c** at −500 mV/SSC_sat._.

**Tab. 2: peak deconvolution parameters of spectra present in Fig. 4.**

| **Potential V/SSC_sat._** | **Peak position (cm^−1^)** | **FWHM (cm^−1^)** | **Area %** | **Assignment** | **References** |
| --- | --- | --- | --- | --- | --- |
| +0.15 (E_corr_) | 419 | 44 | 29 | ν_5_-HSO_4_^−^ | ^22^ |
|  | 444 | 37 | 18 | E_g_-rutile | ^3–5^ |
|  | 586 | 46 | 50 | ν _4_-HSO_4_^−^ | ^7–9^ |
|  | 614 | 14 | 3 | A_1g_-rutile | ^3–5^ |
| −0.4 | 420 | 46 | 56 | ν _5_-HSO_4_^−^ | ^22^ |
|  | 586 | 45 | 44 | ν _4_-HSO_4_^−^ | ^7–9^ |
| −0.5 | 391 | 34 | 23 | TiOSO_4_ | ^20,21^ |
|  | 419 | 17 | 4 | ν _5_-HSO_4_^−^ | ^22^ |
|  | 439 | 30 | 29 | ν _2_-SO_4_^2−^ doublet | ^23^ |
|  | 465 | 25 | 17 | ν _2_-SO_4_^2−^ doublet | ^23^ |
|  | 584 | 32 | 15 | ν _4_-HSO_4_^−^ | ^7–9^ |
|  | 612 | 30 | 8 | ν _4_-SO_4_^2−^ triplet | ^23^ |
|  | 641 | 21 | 3 | ν _4_-SO_4_^2−^ triplet | ^23^ |
|  | 655 | 8 | 1 | ν _4_-SO_4_^2−^ triplet | ^23^ |
| −0.6 | 387 | 26 | 14 | TiOSO_4_ | ^20,21^ |
|  | 419 | 26 | 6 | ν _5_-HSO_4_^−^ | ^22^ |
|  | 437 | 25 | 19 | ν _2_-SO_4_^2−^ doublet | ^23^ |
|  | 461 | 31 | 22 | ν _2_-SO_4_^2−^ doublet | ^23^ |
|  | 586 | 37 | 16 | ν _4_-HSO_4_^−^ | ^7–9^ |
|  | 615 | 32 | 10 | ν _4_-SO_4_^2−^ triplet | ^23^ |
|  | 646 | 33 | 9 | ν _4_-SO_4_^2−^ triplet | ^23^ |
|  | 676 | 34 | 4 | ν _4_-SO_4_^2−^ triplet | ^23^ |

**Fig. 5: Raman spectra describing the 300-800 cm^−1^ frequency region.**





**a** Raman spectrum acquired at E_corr_. **b** Raman spectrum acquired at −0.4 V/SSC_sat._. **c** Raman spectrum acquired at −0.5 V/SSC_sat._. **d** Raman spectrum acquired at −0.6 V/SSC_sat._.

**Fig. 6: Raman spectrum describing the 100-300 cm^−1^ frequency region.**

**
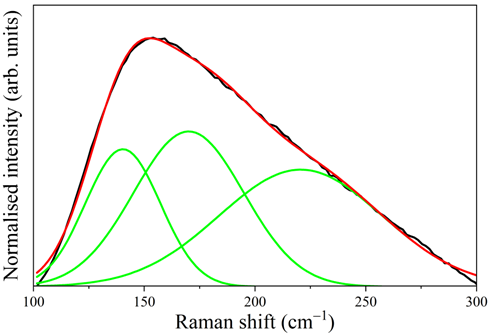
**

Raman spectrum collected over Au substrate at E_corr_ in 40 %v/v H_2_SO_4_.

**Fig. 7: preliminary Raman analysis in the 0-1000 cm^−1^ frequency region.**

**
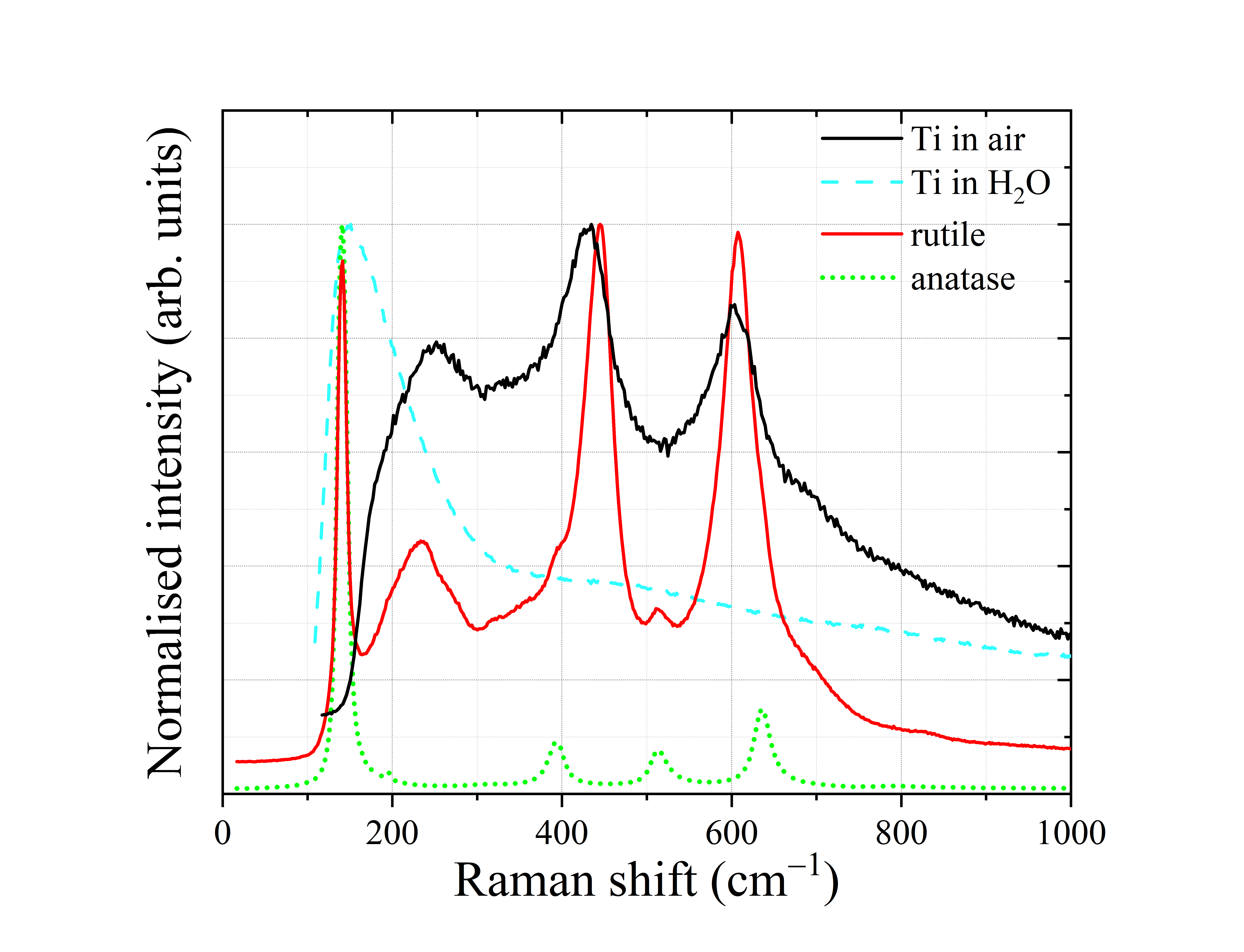
**

Raman spectrum of Ti in water (dashed light blue line), Ti in air (doted-dashed black line), anatase (dotted violet) and rutile (continous red) microparticles.

**Tab. 3: peak deconvolution parameters of spectra present in Fig. 2.**

| **Potential V/SSC_sat._** | **Peak position (cm^−1^)** | **FWHM (cm^−1^)** | **Area %** | **Assignment** | **References** |
| --- | --- | --- | --- | --- | --- |
| +0.15 (E_corr_) | 136 | 19 | 11 | symm H_2_O | ^11–13^ |
|  | 152 | 30 | 23 | asym H_2_O | ^11–13^ |
|  | 183 | 51 | 39 | TiO_6_·H_2_O | ^15,16^ |
|  | 230 | 65 | 27 | Rutile-2^nd^ order scattering | ^3–5^ |
| −0.4 | 136 | 19 | 11 | symm H_2_O | ^11–13^ |
|  | 152 | 30 | 28 | asym H_2_O | ^11–13^ |
|  | 183 | 51 | 46 | TiO_6_·H_2_O | ^15,16^ |
|  | 218 | 27 | 9 | H^+^ insertion | ^16^ |
|  | 248 | 23 | 3 | transl/rotat H_2_O | ^11–13^ |
|  | 274 | 32 | 3 | H^+^ insertion | ^15,17–19^ |
| −0.5 | 137 | 18 | 5 | symm H_2_O | ^11–13^ |
|  | 152 | 32 | 21 | asym H_2_O | ^11–13^ |
|  | 192 | 59 | 63 | TiOSO_4_ | ^20,21^ |
|  | 218 | 23 | 5 | H^+^ insertion | ^16^ |
|  | 249 | 21 | 4 | transl/rotat H_2_O | ^11–13^ |
|  | 276 | 23 | 2 | H^+^ insertion | ^15,17–19^ |
| −0.6 | 135 | 18 | 6 | symm H_2_O | ^11–13^ |
|  | 152 | 30 | 21 | asym H_2_O | ^11–13^ |
|  | 192 | 47 | 55 | TiOSO_4_ | ^20,21^ |
|  | 218 | 17 | 5 | H^+^ insertion | ^16^ |
|  | 248 | 43 | 12 | transl/rotat H_2_O | ^11–13^ |
|  | 275 | 10 | 1 | H^+^ insertion | ^15,17–19^ |

**Tab. 4: Raman peaks water collected on a pure gold substrate at different potentials.**

| **Potential V/SSC_sat._** | **Peak position (cm^−1^)** | **Assignment** | **References** |
| --- | --- | --- | --- |
| +0.15 (E_corr_) | 145 | symm H_2_O | ^11–13^ |
|  | 164 | asym H_2_O | ^11–13^ |
|  | 231 | transl/rotat H2O | ^11–13^ |
| −0.4 | 145 | symm H_2_O | ^11–13^ |
|  | 164 | asym H_2_O | ^11–13^ |
|  | 232 | transl/rotat H2O | ^11–13^ |
| −0.5 | 145 | symm H_2_O | ^11–13^ |
|  | 164 | asym H_2_O | ^11–13^ |
|  | 231 | transl/rotat H2O | ^11–13^ |
| −0.6 | 145 | symm H_2_O | ^11–13^ |
|  | 164 | asym H_2_O | ^11–13^ |
|  | 230 | transl/rotat H2O | ^11–13^ |

**Fig. 8: high frequency portion of the Nyquist representation of Fig. 1d-e.**


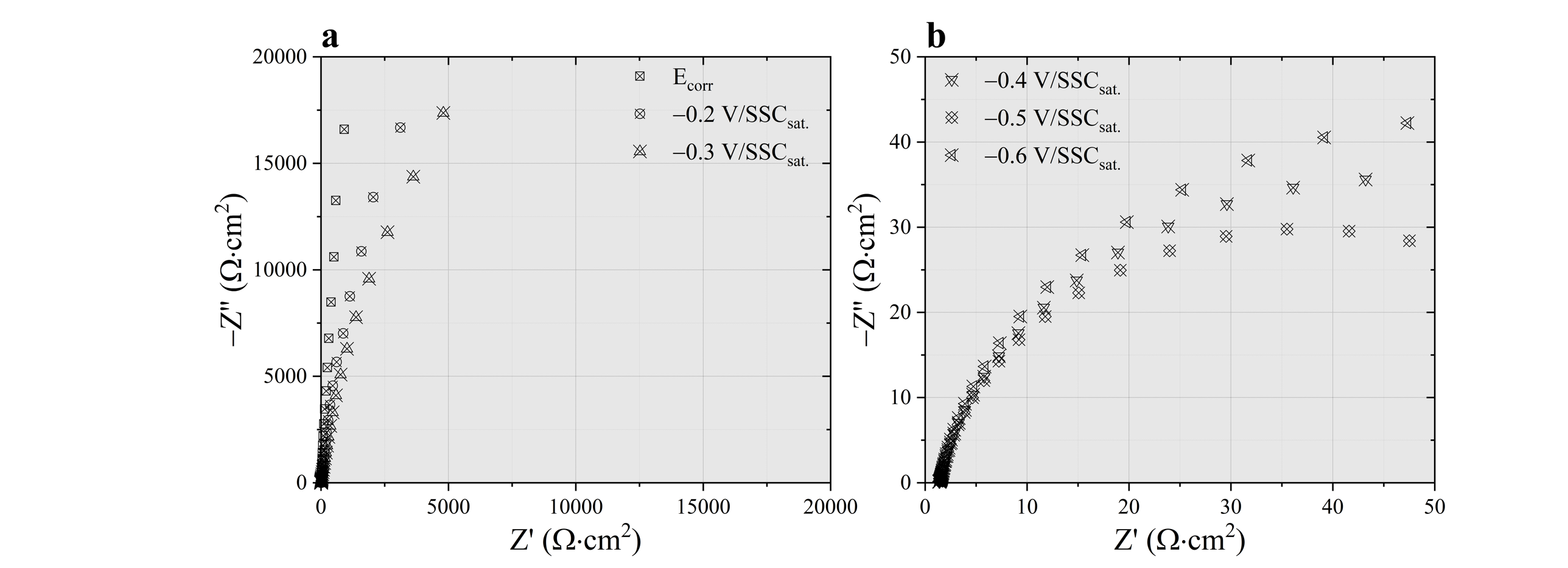


Magnification of the high frequency region of the Nyquist plot highlighted in Fig. 1e-d.

Raman spectroscopy was used to get insight about the chemical modification occurring upon the electrification of the electrode interface. The substrate was characterised according to Raman acquisition performed in air and the main peaks compared with the main lines belonging to pure anatase and rutile micro powders (Sigma-Aldrich). Two evidences can be adduced when looking at **Fig. 7**, *firstly* the polycrystalline sample mainly resembles the spectrum of rutile, clearly showing three distinct features corresponding to the multi-phonon transition around 234 cm^−1^, the E_g_ mode at 432 cm^−1^ and the A_1g_ at 607 cm^−1 29^; *secondly*, when the sample is immersed in the aqueous solution only a distinct features appears at low frequencies corresponding to the intermolecular translational vibration of water^11–13^ while all the other peaks are descaled and almost no more discernible. Moreover, a clear shift of 14 cm^−1^ of the E_g_-rutile peak located at 446 cm^−1^ resulted when analysing the polycrystalline sample, relating this deviation to difference in size of the crystallite contributing to the signal^30^. Prior to consider the spectra collected from light reflection at the Ti substrate immersed in H_2_SO_4_, confidence about the main peaks related to speciation of sulphuric acid, i.e., HSO_4_^−^ and SO_4_^2−^ was reached according to several trials carried out considering a pure gold substrate immersed in different concentration of sulphuric acid and sodium sulphate alone. Four lines belonging to the bisulphate ion were found at 420, 588, 896 and 1036 cm^−1 22,26,28^. The latter one was always the most intense, related to the ν_1_ mode of HSO_4_^−^ paired with H_3_O^+^. Only the intense ν_1_ mode related to sulphates was present in the spectrum at 984 cm^−1 26,28^. A similar approach was carried out considering a sodium sulphate solution with a concentration of 250 g/l and results related to the deconvolution of peaks 448 ˗ ν_2_-SO_4_^2−^ ˗, 615 ˗ ν_4_-SO_4_^2−^ ˗, 978 ˗ ν_1_-SO_4_^2−^ ˗, 1110 cm^−1^ ˗ ν_3_-SO_4_^2−^ ˗ ^23,25^ collected in **Tab. 2**.

Peak deconvolution was performed considering Gaussians for the deconvolution of solid related lines and Gaussian-Lorentzian shapes for the peaks related to aqueous species^31,32^.

The Raman spectrum of the sample exposed to air is very similar to the one collected on pure rutile micro particles. When the sample was immersed in the test solution, the observation of the 2^nd^ order peak located at ~ 230 cm^−1 1,2^ E_g_-rutile^3–5^ (444 cm^−1^) and A_1g_-rutile^3–5^ (614 cm^−1^), appearing near the ν_5_-HSO_4_^−^ (~ 419 cm^−1^)^6^ and ν_4_-HSO_4_^−^ (~ 586 cm^−1^)^7–9^, is a confirmation that the immersion in sulphuric acid did not alter immediately the crystal structure at E_corr_, or even induce the rutile to anatase phase transition observed by Yamazaki *et al.*^10^. Moreover, the low frequency region of the spectrum appeared dominated by the intermolecular translational vibrations of water^11–13^, whose modes are now accepted to be related to hydrogen bonding and additional effects pertaining to the surrounding environment^13,14^, resulting in three peaks describing symmetric (~ 136 cm^−1^), antisymmetric (~ 152 cm^−1^) stretching vibration of H bonds and coupled translation/rotation mode (~ 248 cm^−1^).

**Fig. 9: electrochemical equivalent circuits.**

**
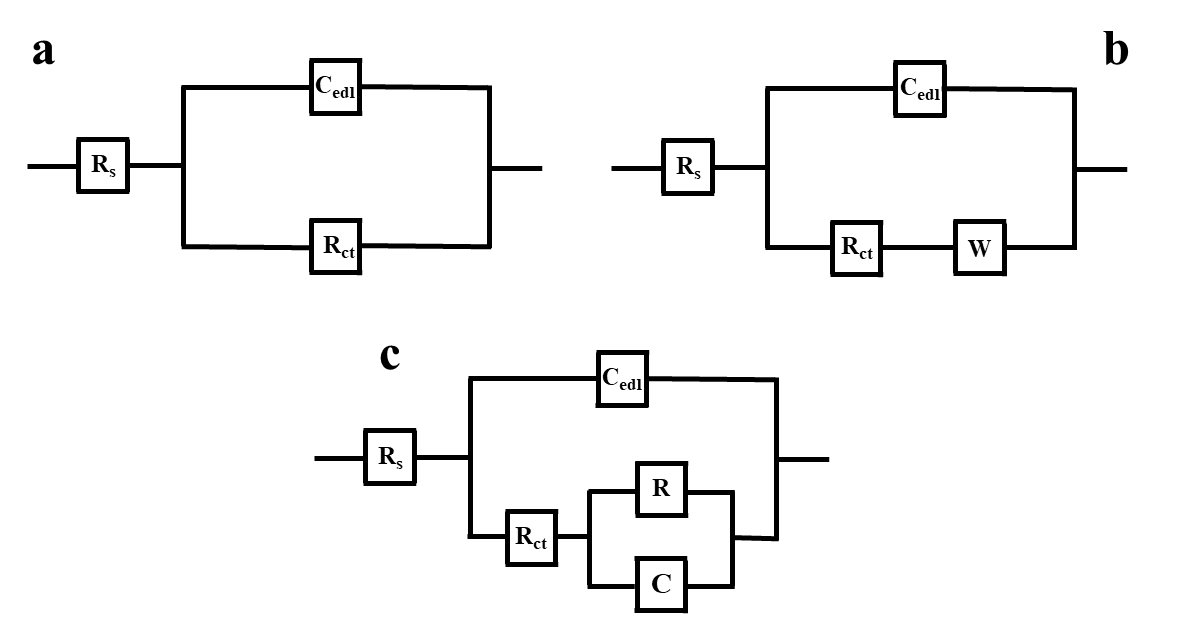
**

**a** Circuit used to fit EIS data at E_corr_. **b** Circuit to fit EIS data accounting for diffusion. **c** Circuit to fit EIS data near the critical potential. *R_s_* refers to the electrolyte resistance, *R_ct_* is the charge transfer resistance, *C_edl_* the capacitance of the double layer, *W* the Warburg element, *R* and *C* the resistance and capacitance associated with the Heyrovsky step of hydrogen evolution.

$\sigma=\frac{RT}{n^{2}F^{2}A\sqrt{2}}\left( \frac{1}{D_{{H_{3}O}^{+}}^{\frac{1}{2}}C_{{H_{3}O}^{+}}^{bulk}} \right)$ (1)

**Eq. 1** describes the Warburg constant $\sigma$ where A (cm^2^) is the geometric surface area, T (K) is temperature, R is the gas constant (J/mol·K), F (C/mol) the Faraday constant, *n*=1 the equivalence of the reaction and $C_{{H_{3}O}^{+}}^{bulk}$(mol/cm^3^) is the proton concentration in the bulk evaluated according to Raman spectroscopy performed over Au. This quantity was calculated as two times the concentration of the sulphates plus the concentration of the bisulphates present in solution. As no variations were encountered during the cathodic sweep a common value of 8.69 M was adopted for all the calculations. **Eq. 2** was used to evaluate the proton diffusion length, where t is the polarisation time.

$l=\sqrt{\pi D_{{H_{3}O}^{+}}t}$ (2)

**Tab. 5: values extracted from EIS data fitting with circuits shown in Fig. 1.**

| **Potential V/SSC_sat._** | **Parameter** | **Value** | **χ^2^** |
| --- | --- | --- | --- |
| +0.15 (E_corr_) | R_s_ (Ω·cm^2^) | 1.51±0.09 | 0.08±0.05 |
|  | R_ct_ (Ω·cm^2^) | 1.09·10^6^±0.26 |  |
|  | n | 0.96±0.03 |  |
|  | Y_o_^CPE^ (S·s^n^/cm^2^) | 2.12·10^−5^±0.13 |  |
|  | C_edl_ (F/cm^2^) | 1.39·10^−5^±0.22 |  |
| −0.2 | R_s_ (Ω·cm^2^) | 1.54±0.12 | 0.04±0.06 |
|  | R_ct_ (Ω·cm^2^) | 1.27·10^5^±0.38 |  |
|  | n | 0.94±0.02 |  |
|  | Y_o_^CPE^ (S·s^n^/cm^2^) | 3.08·10^−5^±0.9 |  |
|  | C_edl_ (F/cm^2^) | 1.68·10^−5^±0.34 |  |
|  | Y_o_^W^ (S·s^0.5^/cm^2^) | 5.75·10^−5^±0.28 |  |
| −0.3 | R_s_ (Ω·cm^2^) | 1.51±0.14 | 0.02±0.02 |
|  | R_ct_ (Ω·cm^2^) | 8.46·10^4^±0.19 |  |
|  | n | 0.92±0.04 |  |
|  | Y_o_^CPE^ (S·s^n^/cm^2^) | 5.37·10^−5^±0.24 |  |
|  | C_edl_ (F/cm^2^) | 2.43·10^−5^±0.13 |  |
|  | Y_o_^W^ (S·s^0.5^/cm^2^) | 8.43·10^−5^±0.35 |  |
| −0.5 | R_s_ (Ω·cm^2^) | 1.41±0.39 | 0.005±0.003 |
|  | R_ct_ (Ω·cm^2^) | 69.01±17 |  |
|  | n | 0.89±0.06 |  |
|  | Y_o_^CPE^ (S·s^n^/cm^2^) | 1.55·10^−4^±0.07 |  |
|  | C_edl_ (F/cm^2^) | 5.73·10^−5^±0.11 |  |
|  | R (Ω·cm^2^) | 701.40±87 |  |
|  | n | 0.93±0.03 |  |
|  | Y_o_^CPE^ (S·s^n^/cm^2^) | 1.55·10^−2^±0.64 |  |
|  | C (F/cm^2^) | 1.17·10^−2^±0.34 |  |
| −0.6 V/SSC_sat._ | R_s_ (Ω·cm^2^) | 1.43±0.23 | 0.004±0.001 |
|  | R_ct_ (Ω·cm^2^) | 103.72±14 |  |
|  | n | 0.88±0.05 |  |
|  | Y_o_^CPE^ (S·s^n^/cm^2^) | 1.23·10^−4^±0.05 |  |
|  | C_edl_ (F/cm^2^) | 4.00·10^−5^±0.12 |  |
|  | R (Ω·cm^2^) | 193.04±31 |  |
|  | n | 0.94±0.02 |  |
|  | Y_o_^CPE^ (S·s^n^/cm^2^) | 1.96·10^−2^±0.13 |  |
|  | C (F/cm^2^) | 1.60·10^−2^±0.21 |  |

**Fig. 10: corrosion product characterisation.**


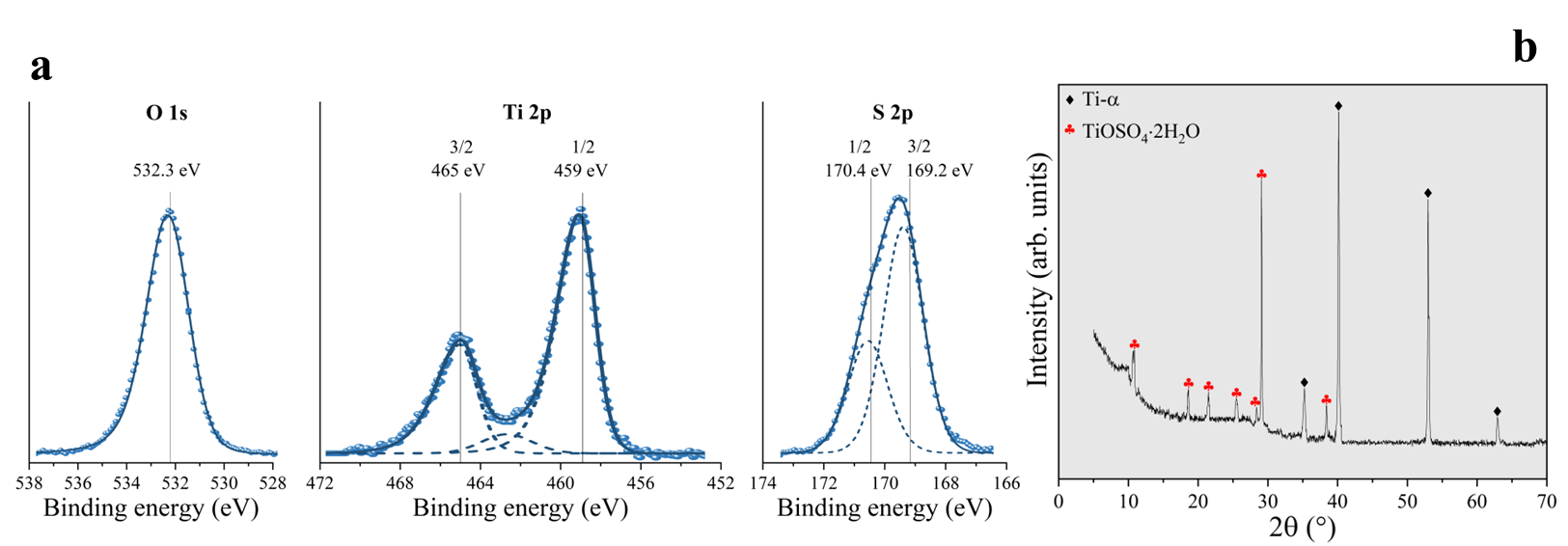


**a** High resolution XPS spectra of O_1s_, Ti_2p_ and S_2p_ components. **b** X-ray diffractogram of TiOSO_4_·2H_2_O.

**Fig. 10a** presents the high-resolution features related to O_1s_, Ti_2p_ and S_2p_. As it is possible to see Ti_2p_ spectrum was dominated by Ti^4+^ components, while the binding energy pertaining to the O_1s_ (532.3 eV) suggests the participation of O in the form of SO_4_^2-^ as it is also confirmed by the binding energy found for the S_2p_ component^10^. X-ray diffraction (**Fig. 10b**) can be used to investigate the crystal lattice of the corrosion products. In particular, working with Bragg-Brentano geometry the film was found to be composed by TiOSO_4_·2H_2_O.

**Tab. 6: peak deconvolution parameters of spectra present in Fig. 4.**

| **Region** | **Peak position (cm^−1^)** | **FWHM (cm^−1^)** | **Area %** | **Assignment** | **References** |
| --- | --- | --- | --- | --- | --- |
| Outside | 886 | 35 | 11 | ν_4_-HSO_4_^−^ | ^7–9^ |
|  | 983 | 33 | 22 | ν_1_-SO_4_^2−^ | ^23–25^ |
|  | 1034 | 44 | 67 | ν_1_-HSO_4_^−^· H_3_O^+^ | ^6,26,27^ |
| Border | 888 | 45 | 9 | ν_4_-HSO_4_^−^ | ^7–9^ |
|  | 991 | 35 | 22 | Ti IV-SO_4_^2−^ (ML_2_) | ^26–28^ |
|  | 1010 | 10 | 3 | Ti IV-SO_4_^2−^ (ML) | ^26–28^ |
|  | 1029 | 24 | 55 | ν_1_-HSO_4_^−^· H_3_O^+^ | ^6,26,27^ |
|  | 1055 | 20 | 7 | ν_1_-HSO_4_^−^ free | ^6,26,27^ |
|  | 1180 | 21 | 4 | ν_3_-SO_4_^2-^ | ^9,23^ |
| Inside | 991 | 31 | 31 | Ti IV-SO_4_^2−^ (ML_2_) | ^26–28^ |
|  | 1010 | 8 | 1 | Ti IV-SO_4_^2−^ (ML) | ^26–28^ |
|  | 1029 | 21 | 62 | ν_1_-HSO_4_^−^· H_3_O^+^ | ^6,26,27^ |
|  | 1055 | 8 | 1 | ν_1_-HSO_4_^−^ free | ^6,26,27^ |
|  | 1181 | 20 | 5 | ν_3_-SO_4_^2−^ | ^9,23^ |

**References**

1. Parker, J. C. & Siegel, R. W. Calibration of the Raman spectrum to the oxygen stoichiometry of nanophase TiO2. *Appl. Phys. Lett.* **57**, 943–945 (1990).

2. Socrates, G. *Infrared and Raman characteristic group frequencies. Tables and charts*. *Journal of Raman Spectroscopy* (2001).

3. Swamy, V., Muddle, B. C. & Dai, Q. Size-dependent modifications of the Raman spectrum of rutile TiO 2. *Appl. Phys. Lett.* **89**, (2006).

4. Nicola, J. H., Brunharoto, C. A., Abramovich, M. & Conçalves da Silva, C. E. T. Second order Raman spectrum of rutile TiO2. *J. Raman Spectrosc.* **8**, 32–34 (1979).

5. Rossella, F. *et al.* TiO2 thin films for spintronics application: A raman study. *J. Raman Spectrosc.* **41**, 558–565 (2010).

6. Turner, D. J. Raman spectral study of bisulphate ion hydration. *J. Chem. Soc. Faraday Trans. 2 Mol. Chem. Phys.* **68**, 643–648 (1972).

7. Querry, M. R. *et al.* Optical constants in the infrared for K2SO4, NH4H2PO4, and H2SO4 in water. *J. Opt. Soc. Am.* **64**, 2–9 (1973).

8. Cox, R. A., Haldna, Ü. L., Idler, K. L. & Yates, K. Resolution of Raman spectra of aqueous sulfuric acid mixtures using principal factor analysis. *Can. J. Chem.* **59**, 2591–2598 (1981).

9. Clegg, S. L. *et al.* Comment on the ‘thermodynamic dissociation constant of the bisulfate ion from Raman and ion interaction modeling studies of aqueous sulfuric acid at low temperatures’. *J. Phys. Chem. A* **109**, 2703–2709 (2005).

10. Yamazaki, S., Fujinaga, N. & Araki, K. Effect of sulfate ions for sol-gel synthesis of titania photocatalyst. *Appl. Catal. A Gen.* **210**, 97–102 (2001).

11. Walrafen, G. E., Chu, Y. C. & Piermarini, G. J. Low-frequency Raman scattering from water at high pressures and high temperatures. *J. Phys. Chem.* **100**, 10363–10372 (1996).

12. Abe, K. & Shigenari, T. Raman spectra of proton ordered phase XI of ICE I. Translational vibrations below 350 cm-1. *J. Chem. Phys.* **134**, (2011).

13. Galvin, M. & Zerulla, D. The extreme low-frequency Raman spectrum of liquid water. *ChemPhysChem* **12**, 913–914 (2011).

14. Tsai, K. H. & Wu, T. M. Local structural effects on low-frequency vibrational spectrum of liquid water: The instantaneous-normal-mode analysis. *Chem. Phys. Lett.* **417**, 389–394 (2006).

15. Tao, G., Fjellvåg, H. & Norby, P. Raman scattering properties of a protonic titanate HxTi 2-x/4□x/4O4·H2O (□, vacancy; x = 0.7) with lepidocrocite-type layered structure. *J. Phys. Chem. B* **112**, 9400–9405 (2008).

16. Denisova, T. A. *et al.* Metatitanic acid: Synthesis and properties. *Russ. J. Inorg. Chem.* **51**, 691–699 (2006).

17. Shin, S. H., Aggarwal, R. L., Lax, B. & Honig, J. M. Raman scattering in Ti2O3-V2O3alloys. *Phys. Rev. B* **9**, 583–590 (1974).

18. Hasegawa, G. *et al.* Hierarchically porous monoliths based on N-doped reduced titanium oxides and their electric and electrochemical properties. *Chem. Mater.* **25**, 3504–3512 (2013).

19. Kukovecz, Á., Hodos, M., Kónya, Z. & Kiricsi, I. Complex-assisted one-step synthesis of ion-exchangeable titanate nanotubes decorated with CdS nanoparticles. *Chem. Phys. Lett.* **411**, 445–449 (2005).

20. Kiyomi, L., Ronil, F. & Sanches, N. Titanyl sulphate, an inorganic polymer: structural studies and vibrational assignment. *Quim. Nov.* **42**, 1112–1115 (2019).

21. Reynolds, M. L. & Wiseman, T. J. Some observations on the structure of titanyl sulphate dihydrate. *J. Inorg. Nucl. Chem.* **29**, 1381–1383 (1967).

22. Turner, D. J. Raman spectral study of bisulphate ion hydration. *J. Chem. Soc. Faraday Trans. 2 Mol. Chem. Phys.* **68**, 643–648 (1972).

23. Ben Mabrouk, K., Kauffmann, T. H., Aroui, H. & Fontana, M. D. Raman study of cation effect on sulfate vibration modes in solid state and in aqueous solutions. *J. Raman Spectrosc.* **44**, 1603–1608 (2013).

24. Chen, H. & Irish, D. E. A raman spectral study of bisulfate-sulfate systems. III. Salt effects. *J. Phys. Chem.* **75**, 2681–2684 (1971).

25. Sharma, S. K. *et al.* Standoff Raman spectroscopy for future Europa Lander missions. *J. Raman Spectrosc.* **51**, 1782–1793 (2020).

26. Baillon, F., Provost, E. & Fürst, W. Study of titanium(IV) speciation in sulphuric acid solutions by FT-Raman spectrometry. *J. Mol. Liq.* **143**, 8–12 (2008).

27. Szilágyi, I., Königsberger, E. & May, P. M. Characterization of chemical speciation of titanyl sulfate solutions for production of titanium dioxide precipitates. *Inorg. Chem.* **48**, 2200–2204 (2009).

28. Wang, W. *et al.* Mechanism and kinetics of titanium hydrolysis in concentrated titanyl sulfate solution based on infrared and Raman spectra. *Chem. Eng. Sci.* **134**, 196–204 (2015).

29. Balachandran, U. & Eror, N. G. Raman spectra of titanium dioxide. *J. Solid State Chem.* **42**, 276–282 (1982).

30. Ekoi, E. J., Gowen, A., Dorrepaal, R. & Dowling, D. P. Characterisation of titanium oxide layers using Raman spectroscopy and optical profilometry: Influence of oxide properties. *Results Phys.* **12**, 1574–1585 (2019).

31. Bradley, M. Key Words • Collision-induced Scattering • OMNIC Peak Resolve Software • Peak Fitting • Protein Structures • Vibrational Line Shapes Curve Fitting in Raman and IR Spectroscopy: Basic Theory of Line Shapes and Applications.

32. Schuster, J. J., Will, S., Leipertz, A. & Braeuer, A. Deconvolution of Raman spectra for the quantification of ternary high-pressure phase equilibria composed of carbon dioxide, water and organic solvent. *J. Raman Spectrosc.* **45**, 246–252 (2014).

33. Wang, Y. H. *et al.* In situ Raman spectroscopy reveals the structure and dissociation of interfacial water. *Nature* **600**, 81–85 (2021).

34. Seki, T. *et al.* The bending mode of water: A powerful probe for hydrogen bond structure of aqueous systems. *J. Phys. Chem. Lett.* **11**, 8459–8469 (2020).

**Raw data**

| 50.44872 | 0 |
| --- | --- |
| 52.21343 | -7.37566E-4 |
| 53.9778 | -8.66912E-4 |
| 55.74184 | 0 |
| 57.5034 | 0 |
| 59.26892 | 6.00124E-4 |
| 61.02982 | 0.00148 |
| 62.79253 | 5.21743E-4 |
| 64.55491 | -6.09768E-5 |
| 66.31696 | -4.4078E-5 |
| 68.07867 | 0 |
| 69.83792 | 0 |
| 71.60112 | -3.19949E-4 |
| 73.3597 | -2.47023E-4 |
| 75.12009 | 2.47051E-4 |
| 76.88016 | 9.5903E-4 |
| 78.63989 | 0.00116 |
| 80.39715 | 5.81716E-4 |
| 82.15836 | 8.86476E-4 |
| 83.91496 | 8.32066E-4 |
| 85.67336 | -9.96995E-4 |
| 87.43144 | -0.00145 |
| 89.18919 | -0.00111 |
| 90.94447 | -4.67125E-4 |
| 92.70369 | -5.41793E-4 |
| 94.45831 | -9.52768E-4 |
| 96.21474 | -5.86267E-4 |
| 97.97084 | 6.2134E-4 |
| 99.7266 | 0.00276 |
| 101.47991 | 0.00593 |
| 103.23715 | 0.00874 |
| 104.98979 | 0.01267 |
| 106.74425 | 0.01891 |
| 108.49837 | 0.02613 |
| 110.25215 | 0.0331 |
| 112.00349 | 0.04238 |
| 113.75875 | 0.05283 |
| 115.50942 | 0.06524 |
| 117.25977 | 0.0782 |
| 119.01405 | 0.09359 |
| 120.76373 | 0.10768 |
| 122.51522 | 0.12272 |
| 124.26638 | 0.13763 |
| 126.01721 | 0.15169 |
| 127.76559 | 0.16423 |
| 129.51576 | 0.17775 |
| 131.26561 | 0.18817 |
| 133.01302 | 0.19786 |
| 134.76221 | 0.20663 |
| 136.51106 | 0.21248 |
| 138.25748 | 0.21672 |
| 140.00569 | 0.22157 |
| 141.75357 | 0.22252 |
| 143.50113 | 0.22388 |
| 145.24623 | 0.22646 |
| 146.99312 | 0.22928 |
| 148.7397 | 0.23044 |
| 150.48383 | 0.23123 |
| 152.22974 | 0.23361 |
| 153.97533 | 0.23114 |
| 155.71848 | 0.23122 |
| 157.46341 | 0.22998 |
| 159.20802 | 0.2232 |
| 160.9502 | 0.22109 |
| 162.69415 | 0.22018 |
| 164.43779 | 0.21696 |
| 166.17897 | 0.21434 |
| 167.92195 | 0.21331 |
| 169.66461 | 0.2112 |
| 171.40482 | 0.20824 |
| 173.14682 | 0.20824 |
| 174.8885 | 0.20415 |
| 176.62773 | 0.19851 |
| 178.36876 | 0.19803 |
| 180.10947 | 0.19587 |
| 181.84984 | 0.19708 |
| 183.58777 | 0.19788 |
| 185.32538 | 0.19925 |
| 187.06689 | 0.2007 |
| 188.80386 | 0.20401 |
| 190.5405 | 0.20526 |
| 192.27892 | 0.20363 |
| 194.01701 | 0.20253 |
| 195.75269 | 0.20215 |
| 197.49013 | 0.19981 |
| 199.22514 | 0.20287 |
| 200.96194 | 0.20189 |
| 202.69843 | 0.20344 |
| 204.43248 | 0.20718 |
| 206.1662 | 0.2103 |
| 207.90381 | 0.21277 |
| 209.63689 | 0.21321 |
| 211.36963 | 0.21414 |
| 213.10417 | 0.21193 |
| 214.83627 | 0.20761 |
| 216.57016 | 0.20278 |
| 218.30373 | 0.19544 |
| 220.03485 | 0.18561 |
| 221.76567 | 0.17429 |
| 223.49826 | 0.16235 |
| 225.23053 | 0.15014 |
| 226.96037 | 0.13736 |
| 228.692 | 0.12568 |
| 230.4212 | 0.11527 |
| 232.15009 | 0.10843 |
| 233.88074 | 0.09955 |
| 235.61107 | 0.09335 |
| 237.33899 | 0.08872 |
| 239.06868 | 0.08672 |
| 240.79594 | 0.08492 |
| 242.52499 | 0.08211 |
| 244.25162 | 0.08038 |
| 245.97792 | 0.07622 |
| 247.7081 | 0.07309 |
| 249.43375 | 0.07114 |
| 251.15909 | 0.06538 |
| 252.8862 | 0.06201 |
| 254.6109 | 0.06035 |
| 256.33527 | 0.05839 |
| 258.06143 | 0.05718 |
| 259.78516 | 0.05484 |
| 261.50858 | 0.05185 |
| 263.23587 | 0.04661 |
| 264.95862 | 0.04222 |
| 266.68109 | 0.0395 |
| 268.4053 | 0.03644 |
| 270.12711 | 0.03326 |
| 271.8486 | 0.03013 |
| 273.57187 | 0.02913 |
| 275.29272 | 0.03071 |
| 277.01535 | 0.03107 |
| 278.73553 | 0.03008 |
| 280.45544 | 0.02852 |
| 282.17709 | 0.02685 |
| 283.89636 | 0.02532 |
| 285.6153 | 0.02392 |
| 287.336 | 0.02132 |
| 289.05429 | 0.01905 |
| 290.77225 | 0.01692 |
| 292.492 | 0.01453 |
| 294.20932 | 0.01194 |
| 295.92844 | 0.00906 |
| 297.64514 | 0.00588 |
| 299.36151 | 0.00242 |
| 301.07965 | -0.00133 |
